# Permanent Oviduct Posteriorization after Neonatal Exposure to the Phytoestrogen Genistein

**DOI:** 10.1289/ehp.1104018

**Published:** 2011-08-02

**Authors:** Wendy N. Jefferson, Elizabeth Padilla-Banks, Jazma Y. Phelps, Kevin E. Gerrish, Carmen J. Williams

**Affiliations:** 1 Reproductive Medicine Group, Laboratory of Reproductive and Developmental Toxicology, and; 2 Microarray Group, National Institute of Environmental Health Sciences, National Institutes of Health, Department of Health and Human Services, Research Triangle Park, North Carolina, USA

**Keywords:** developmental patterning, female reproductive tract, genistein, homeobox gene

## Abstract

Background: Preimplantation embryo loss during oviduct transit has been observed in adult mice after a 5-day neonatal exposure to the phytoestrogen genistein (Gen; 50 mg/kg/day).

Objective: We investigated the mechanisms underlying the contribution of the oviduct to infertility.

Methods: Female mice were treated on postnatal days 1–5 with corn oil or Gen (50 mg/kg/day). We compared morphology, gene expression, and protein expression in different regions of the reproductive tracts of Gen-treated mice with those of control littermates at several time points.

Results: Neonatal Gen treatment resulted in substantial changes in expression of genes that modulate neonatal oviduct morphogenesis, including *Hoxa* (homeobox A cluster), *Wnt* (wingless-related MMTV integration site), and hedgehog signaling genes. An estrogen receptor antagonist blocked these effects, indicating that they were induced by the estrogenic activity of Gen. Oviducts of adults treated neonatally with Gen had abnormal morphology and were stably “posteriorized,” as indicated by altered *Hoxa* gene patterning during the time of treatment and dramatic, permanent up-regulation of homeobox genes (e.g., *Pitx1*, *Six1*) normally expressed only in the cervix and vagina.

Conclusions: Neonatal exposure to estrogenic environmental chemicals permanently disrupts oviduct morphogenesis and adult gene expression patterns, and these changes likely contribute to the infertility phenotype.

The effects of endocrine-disrupting chemicals critically depend on the developmental timing of exposure and the concentration of active chemical that actually reaches the target tissue. Exposure to estrogenic chemicals is a well-documented risk to human health based on the experience with prenatal exposure to diethylstilbestrol (DES) and its effects on development of the female reproductive tract (FRT). Many estrogenic compounds are found as environmental contaminants at levels sufficient to have adverse effects on human health. Phytoestrogens in the diet or nutritional supplements are common sources of environmental estrogen exposure (Reinli and Block 1996). The most abundant dietary phytoestrogens are the isoflavones genistein (Gen) and daidzein, which are present in soybean-derived products (Reinli and Block 1996). Even in strict vegetarians, adult serum isoflavone levels are low (≤ 0.5 μM) and should have minimal estrogenic activity (Jefferson 2010; Peeters et al. 2007).

In contrast to adults, infants fed soy-based formula have relatively high serum isoflavone concentrations; in a recent study, Cao et al. (2009) found that the 25th, 50th, and 75th percentiles in serum were 1.5, 3.3, and 5.4 μM, respectively, and the highest concentration was 13.2 μM. In animal models these levels are clearly estrogenic, as indicated by uterine wet weight bioassays (Jefferson and Williams 2010). Two prospective studies demonstrate that soy-based infant formula elicits estrogenic responses in the developing human female (Bernbaum et al. 2008; Zung et al. 2008). Limited epidemiological information is available regarding long-term effects of infant exposure to soy-based formulas, but in a recent large study, D’Aloisio et al. (2010) reported an increased incidence of physician-diagnosed fibroids by 35 years of age. These studies suggest that neonatal exposure to phytoestrogens or other environmental estrogenic chemicals could have a long-term effect on adult female reproductive health.

FRT development is similar in most mammals, although the timing of morphogenesis differs among species. In the mouse, the FRT develops from the Müllerian duct beginning on gestation day (GD) 13 (Masse et al. 2009). Prenatal FRT development is controlled by several transcription factors and secreted signaling glycoproteins of the Wnt (wingless-related MMTV integration site) and hedgehog families. Anterior–posterior (AP) patterning of the Müllerian duct is regulated by positional information derived from the Hoxa (homeobox A cluster) family of homeobox transcription factors. Expression of these genes becomes spatially restricted before birth, and distinct FRT regions eventually develop that correlate with increased expression of specific Hoxa genes: *Hoxa9* in the oviduct, *Hoxa10* and *Hoxa11* in the uterus and upper cervix, and *Hoxa13* in the lower cervix and vagina (Block et al. 2000; Ma et al. 1998; Taylor et al. 1997).

Epithelial cell differentiation in the mouse FRT begins after birth and continues until adulthood. At birth, the oviduct is a simple tubular structure with a single layer of columnar epithelium. During the neonatal period, this epithelium differentiates into secretory or ciliated columnar cells in response to cues received from the underlying mesenchyme that vary along the AP axis (Yamanouchi et al. 2010). These differences result in distinct patterns of epithelial morphogenesis such that the oviduct infundibulum and ampulla consist largely of ciliated epithelial cells, whereas the isthmus has more secretory cells. Similarly, uterine and vaginal mesenchymal cells generate cues that determine the epithelial cell types in those regions, with the adult uterus having mainly simple columnar cells and the vagina developing a stratified squamous epithelium (Kurita et al. 2001).

The neonatal FRT is highly sensitive to estrogenic disruption during morphogenesis and cellular differentiation. Numerous studies performed in animal models show that prenatal or neonatal exposure to estrogenic compounds causes dramatic alterations in FRT development and can lead to FRT cancers (Ma 2009). To determine whether exposure to environmentally relevant phytoestrogens has similar effects on FRT development and function, we used a mouse model of subcutaneous Gen exposure on postnatal days (PND) 1–5 (50 mg/kg/day). As long as the final serum Gen concentration is similar, the effects of Gen derived from oral exposure during the neonatal period are comparable to those observed when Gen is administered subcutaneously (Jefferson et al. 2009a). This model generates serum Gen levels [maximum concentration (*C*_max_) in female pups, 6.8 μM] similar to those found in human infants fed soy-based infant formula (Cao et al. 2009; Doerge et al. 2002). As adults, these mice have multioocyte follicles, lack regular estrous cycles, and are infertile even after superovulation, and approximately 30% develop uterine cancer (Jefferson et al. 2005; Newbold et al. 2001). Abnormalities in both the oviduct and uterus contribute to the infertility phenotype. Although the mice become pregnant, approximately 50% of the preimplantation embryos die in the oviduct after the two-cell stage, and the uterus does not support implantation (Jefferson et al. 2009b). The goal of the present study was to test the hypothesis that neonatal Gen treatment causes permanent changes in oviduct gene expression and morphology that could explain the oviduct’s contribution to infertility.

## Materials and Methods

*Animals and treatments.* All animal procedures complied with National Institutes of Health/National Institute of Environmental Health Sciences animal care guidelines; animals were treated humanely and with regard for alleviation of suffering. Mice were fed NIH-31 mouse chow (Zeigler Brothers, Gardners, PA), which was assayed for phytoestrogen content as previously described (Jefferson et al. 2009b). Female CD-1 pups were injected subcutaneously on PND1–PND5 with corn oil (control) or Gen (50 mg/kg/day; Sigma, St. Louis, MO), ICI-182780 (ICI; 1 mg/kg/day; Sigma), or ICI (1 mg/kg/day) 30 min before Gen (50 mg/kg/day). Gen was prepared in a suspension of corn oil at 5 mg/mL, and ICI was dissolved in corn oil at 1 mg/mL. All treatments were delivered in 20 μL corn oil, and doses were calculated based on a pup weight of 2.0 g. At 6–8 weeks of age, the female mice underwent superovulation [equine chorionic gonadotropin followed by human chorionic gonadotropin (hCG), both at 5.0 IU in 0.1 mL saline; both from Calbiochem, Gibbstown, NJ] or superovulation and mating as described previously (Jefferson et al. 2009b). Only plug-positive mated females were used in experiments requiring pregnant mice.

*Real-time reverse-transcriptase polymerase chain reaction (RT-PCR).* Samples were collected as follows (*n* = 3–6 independent biological replicates per group). On PND1, oviducts, uteri, and cervix/vagina from five mice were pooled per biological replicate. On PND5, oviducts, uteri, and cervix/vagina were collected 4 hr after the last treatment; oviducts from three mice were pooled per biological replicate, but uterus and cervix/vagina samples were from individual mice. For PND22 pups and adults, both oviducts from one mouse were pooled per biological replicate. For adult nonpregnant mice, oviducts of superovulated females were collected before (8 hr after hCG) and after (15 hr after hCG) ovulation, and cumulus masses were removed. For adult pregnant mice, oviducts of superovulated females were collected on GD2 or GD4 (48 hr or 96 hr after hCG, respectively), and embryos were removed. For untreated adult FRT samples, oviducts (pair), uterus, cervix, and vagina collected from one mouse per biological replicate.

Total RNA was isolated, and cDNA was generated from 1 μg RNA using standard protocols. Primers were designed to amplify exon junctions. RT-PCR was performed on 20 ng cDNA using SYBR_®_ Green–based detection on an ABI 7200 HT sequence detector (Applied Biosystems, Foster City, CA). Relative gene expression was calculated by the ΔC_t_ method (Pfaffl 2001) using cyclophilin A (*Ppia*) expression for normalization.

*Microarray analysis.* Oviducts were collected on GD2 (48 hr after hCG), and embryos were removed. RNA was isolated using the RNeasy Mini Kit (Qiagen, Valencia, CA). Gene expression analysis was conducted on four independent biological replicates for each group using Agilent Whole Mouse Genome 4 × 44 multiplex format oligo arrays (Agilent Technologies, Santa Clara, CA). Slides were scanned with an Agilent Scanner, and data were obtained using Agilent Feature Extraction software (version 9.5), which performed error modeling, adjusting for additive and multiplicative noise. The resulting data were processed using Rosetta Resolver_®_ (version 7.2; Rosetta Biosoftware, Kirkland, WA) and Ingenuity Pathway Analysis (Ingenuity Systems, Redwood City, CA) and deposited in the National Center for Biotechnology Information (NCBI) Gene Expression Omnibus (accession no. GSE27639; NCBI, Bethesda, MD).

*Histology and immunohistochemistry.* Formalin-fixed tissues were paraffin embedded and sectioned at 5 μm. Sections were stained with hematoxylin and eosin (H&E) or Masson’s trichrome according to standard protocols. For Pitx1 (paired-like homeodomain transcription factor 1) immunohistochemistry, sections were blocked with 5% bovine serum albumin (BSA) in Tris-buffered saline/Tween 20 (TBST). Rabbit polyclonal anti-Pitx1 antibody (Tremblay et al. 1998) was diluted 1:150 in TBST containing 1% BSA and 1% milk. Biotinylated anti-rabbit IgG (1:500; Vector Laboratories, Burlingame, CA), ExtrAvidin (1:50; Sigma), and NovaRED (Vector) were used for detection. For E-cadherin immunohistochemistry, sections were blocked with 10% horse serum and avidin/biotin blocking reagent (Vector), and E-cadherin antibody (Sigma) was used at a 1:150 dilution. The secondary antibody was from the Vectastain Elite Kit (Vector), and diaminobenzidine was used for visualization.

*Immunoblotting.* Total oviduct protein (5 μg/mouse in each lane) was separated on Novex 4–12% Tris-glycine gels and transferred to polyvinyl difluoride (Invitrogen, Carlsbad, CA). Blots were blocked with 5% milk in TBST and then incubated with either Six1 [sine oculis-related homeobox 1 homolog (Drosophila); 1:1,000; Abcam, Cambridge, UK] or β-actin (0.2 μg/mL; Sigma) primary antibodies followed by horseradish peroxidase–conjugated anti-rabbit or anti-mouse IgG (Amersham, Piscataway, NJ). Bands were visualized using West Femto Reagents (Pierce, Rockford, IL).

*Statistical analysis.* We analyzed data using JMP software (version 9; SAS Institute Inc., Cary, NC). Student’s *t*-tests were used to compare control and Gen-treated samples at each time point.

## Results

**Figure 1:**
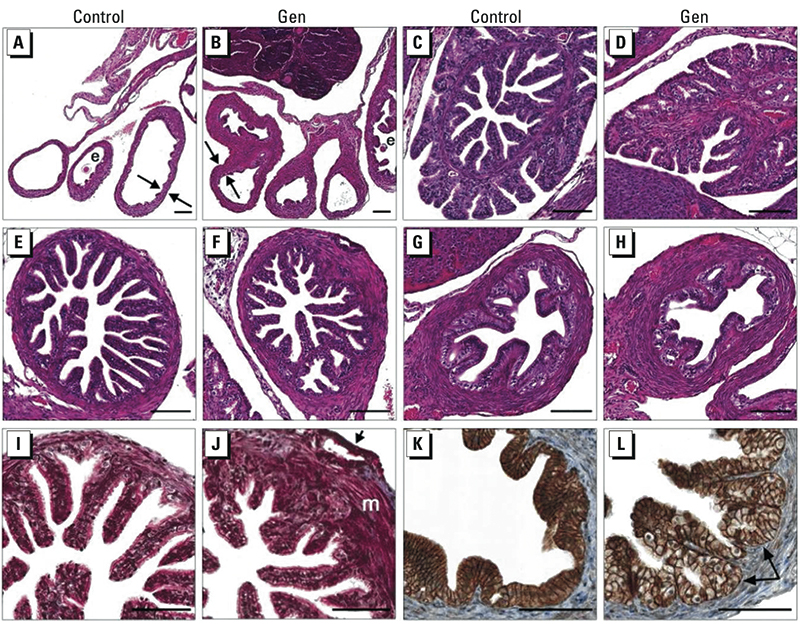
Oviduct histology on GD2 in controls (*A*,*E*,*I*,*C*,*G*,*K*) and after Gen treatment (*B*,*F*,*J*,*D*,*H*,*L*). (*A *and* B*) Low-power view of H&E-stained oviducts; arrows highlight muscle/stroma thickness, and embryos are indicated (e). (*C*–*H*) H&E-stained oviducts showing typical morphology of infundibulum (*C *and* D*), ampulla (*E *and* F*), and isthmus (*G *and* H*). (*I *and* J*) Masson’s trichrome stain, with thickened muscle (m) and pseudogland (arrow) indicated. (*K *and* L*) E-Cadherin immunohistochemistry (brown stain), with arrows indicating pseudoglands. Bars = 100 μm.

*Abnormal oviduct histology after neonatal Gen treatment.* As a first step in determining why preimplantation embryos from neonatal Gen-treated mice had poor survival in the oviduct after the two-cell stage, we analyzed the histology of oviducts collected on GD2. Oviducts from Gen-treated mice retained the expected regional differences between the infundibulum, ampulla, and isthmus (Figure 1A–H); however, significant abnormalities were present in tissue and cellular morphology compared with controls. The stromal and muscle compartments were abnormally thickened in all oviduct regions, reminiscent of uterine myometrium and stroma (Figure 1A–H). The thickened areas mainly consisted of additional muscle tissue, and many areas did not have the normal clearly defined outer and inner smooth muscle layers (Figure 1I,J). In some areas of the ampulla, pseudoglands were near the outer epithelial surface of the oviduct, with no intervening muscle layers (Figure 1J). There were localized areas of excessive epithelial proliferation, and many regions had areas of epithelial disorganization that were particularly apparent after cell borders were labeled by immunostaining for E-cadherin (Figure 1K,L). This staining also revealed that the epithelial cells had enlarged, rounded nuclei and were cuboidal rather than the predominant tall columnar epithelial cells observed in controls.

**Figure 2:**
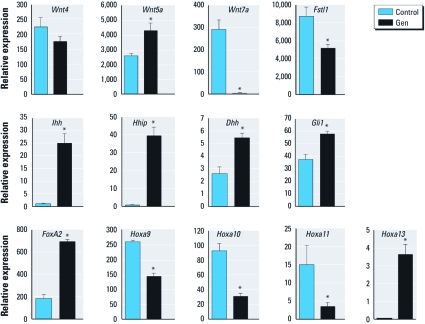
Expression of genes important for FRT development on PND5 after Gen treatment. Data are mean ± SE relative oviduct mRNA expression plotted for indicated genes. *p < 0.05 compared with control.

*Alterations in oviduct gene expression during neonatal Gen treatment.* We hypothesized that the changes in oviduct morphology were a result of Gen-induced alterations in expression of genes that influence postnatal FRT morphogenesis. The effect of Gen treatment on oviduct expression of secreted signaling proteins and transcription factors previously documented to affect FRT development was examined by real-time PCR at the completion of Gen treatment on PND5 (Figure 2). Wnt genes required for FRT development include *Wnt4*, *Wnt5a*, and *Wnt7a* (Masse et al. 2009). There was no difference in *Wnt4* expression, whereas *Wnt5a* was up-regulated and *Wnt7a* was almost completely repressed in Gen-treated mice compared with controls. Follistatin-like-1 (Fstl1), a diffusible oviduct mesenchymal factor, which promotes formation of ciliated epithelial cells (Yamanouchi et al. 2010), was significantly reduced in Gen-treated mice compared with controls. Secreted proteins in the hedgehog signaling pathway can regulate postnatal FRT morphogenesis and adult FRT function, and Wnt genes can be targets of hedgehog signaling (Franco et al. 2010). Indian hedgehog (*Ihh*) and hedgehog interacting protein (*Hhip*) were highly up-regulated, and desert hedgehog (*Dhh*) was up-regulated 2-fold, after Gen treatment compared with controls, whereas sonic hedgehog (*Shh*) was not detected (data not shown). GLI-Kruppel family member 1 (*Gli1*), a common downstream mediator of hedgehog signaling, was also up-regulated, indicating that Gen treatment caused activation of hedgehog signaling in the neonatal oviduct.

Transcription factors that regulate patterning and differentiation of the FRT include forkhead box a2 (*Foxa2*) and the posterior Hoxa genes (Jeong et al. 2010). *Foxa2* expression was up-regulated on PND5 by Gen treatment. Expression of *Hoxa9*, *Hoxa10*, and *Hoxa11* was decreased in oviducts of Gen-treated mice compared with controls, whereas *Hoxa13* was highly up-regulated. This Hoxa gene expression pattern indicated that the PND5 oviduct was posteriorized; that is, the oviduct expressed Hoxa genes in a pattern similar to that normally observed in the posterior cervical and vaginal tissues (Ma et al. 1998).

*Alterations in oviduct gene expression on GD2.* We hypothesized that genes important for FRT morphogenesis and perturbed by neonatal Gen treatment would continue to be altered in adult oviducts and that additional oviduct genes would be misexpressed around the time of preimplantation embryo loss. Rather than choosing candidate genes, we performed a microarray-based gene expression analysis of oviducts from control and Gen-treated mice on GD2. We validated selected probes that covered a range of intensity values and fold changes by real-time RT-PCR [see Supplemental Material, Table 1 (http://dx.doi.org/10.1289/ehp.1104018)]. Principal component analysis showed sample separation into distinct groups based on treatment; this was confirmed using hierarchical cluster analysis (see Supplemental Material, Figure 1).

**Table 1:** Most highly up- and down-regulated oviduct genes.

Gene	Gene name	Fold change (gene/control)
Up-regulated genes				
*Pitx1*		paired-like homeodomain transcription factor 1		99.3
*Serpina3a*		serine (or cysteine) peptidase inhibitor, clade A, member 3A		10.3
*Hao3*		hydroxyacid oxidase (glycolate oxidase) 3		8.4
*Lipf*		lipase, gastric		7.6
*Osap*		ovary-specific acidic protein		7.5
*Igkv5-43*		immunoglobulin kappa chain variable 5-43		7.4
*Prss35*		protease, serine, 35		7.1
*Cxcl15*		chemokine (C-X-C motif) ligand 15		6.9
*Six1*		sine oculis-related homeobox 1 homolog (Drosophila)		6.8
*Serpina5*		serine (or cysteine) peptidase inhibitor, clade A, member 5		6.8
*Cyp11a1*		cytochrome P450, family 11, subfamily a, polypeptide 1		6.7
*Nkx3-1 *		NK-3 transcription factor, locus 1 (Drosophila)		5.9
Down-regulated genes				
*Kcne1*		potassium voltage-gated channel, Isk-related subfamily, member 1		–4.9
*Serpina11*		serine (or cysteine) peptidase inhibitor, clade A, member 11		–4.9
*Myh7*		myosin, heavy polypeptide 7, cardiac muscle, beta		–5.1
*Myh6*		myosin, heavy polypeptide 6, cardiac muscle, alpha		–5.2
*Coch*		Cochlin, coagulation factor C homolog coagulation factor C homolog (Limulus polyphemus)		–5.3
*Serpina1d*		serine (or cysteine) peptidase inhibitor, clade A, member 1D		–5.7
*Olah*		oleoyl-ACP hydrolase		–5.9
*Spock1*		sparc/osteonectin, cwcv and kazal-like domains proteoglycan 1		–6.0
*Serpina1e*		serine (or cysteine) peptidase inhibitor, clade A, member 1E		–6.2
*Serpina1b*		serine (or cysteine) preptidase inhibitor, clade A, member 1B		–6.2
*Epha6*		Eph receptor A6		–7.8
*Ano2*		anoctamin 2		–18.4

Of the > 41,000 probes on the array, 1,125 probes were significantly altered (analysis of variance, *p* < 0.01) in Gen-treated mice compared with controls. Duplicate probes and probes with average intensity < 100 in both treatment groups were removed from the analysis data set. Within the remaining probes, 335 genes were up- or down-regulated ≥ 2-fold; the most highly altered genes are shown in Table 1. Significantly altered Ingenuity biological function categories included development, immune response, and cell proliferation [see Supplemental Material, Table 2 (http://dx.doi.org/10.1289/ehp.1104018)]. Four of the six secreted signaling proteins (*Wnt7a*, *Ihh*, *Dhh*, and *Hhip*) and one transcription factor (*Foxa2*) that were altered on PND5 after Gen treatment by PCR analysis were also significantly altered on GD2 by microarray analysis. Taken together, these findings indicate that neonatal Gen treatment permanently altered gene expression in the adult oviduct.

**Figure 3:**
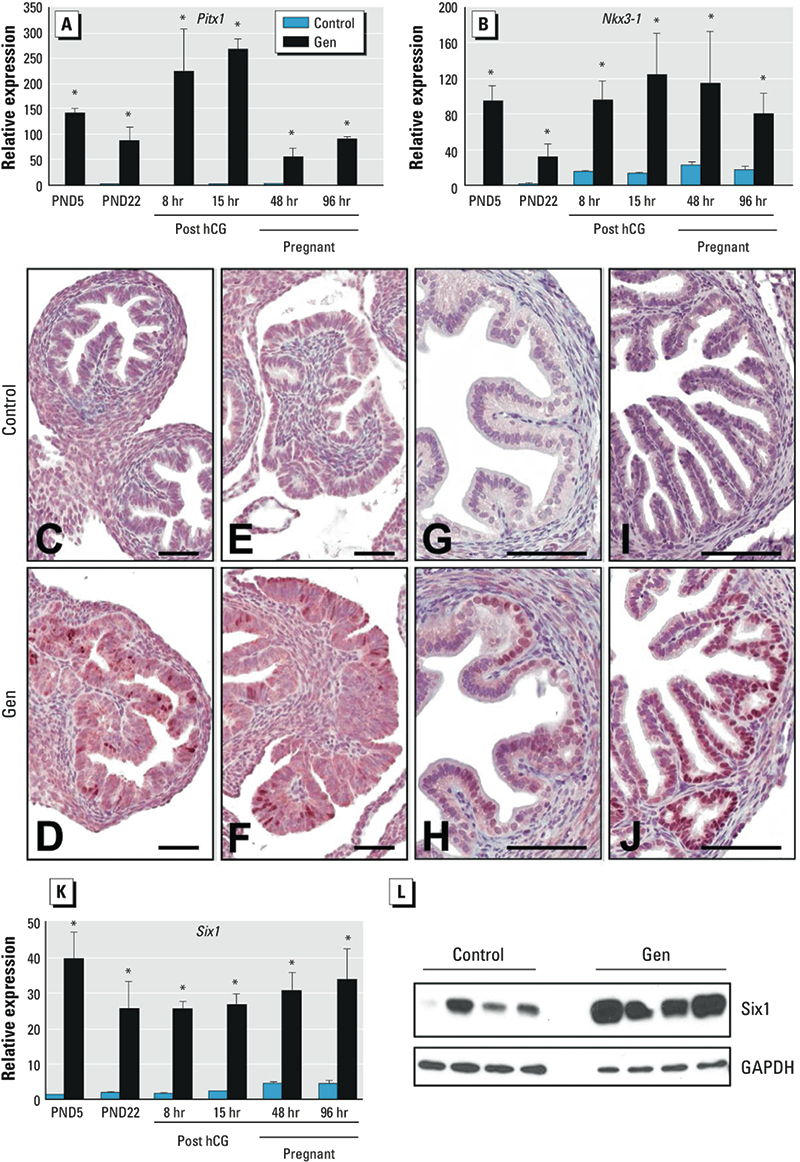
Expression of *Pitx1*, *Nkx3-1*, and *Six1* in oviduct after neonatal Gen treatment. (*A* and *B*) Relative expression (mean ± SE) of *Pitx1* (*A*) and *Nkx3-1* (*B*). (*C*–*J*) Pitx1 immunohistochemistry in PND5 isthmus (*C *and *D*) and ampulla/infundibulum (*E *and *F*) and GD2 isthmus (*G *and *H*) and ampulla (*I *and *J*). Bars = 100 μm. (*K*)**Relative expression (mean ± SE) of *Six1*. (*L*) Immunoblot of Six1 in oviduct on GD2 (protein from one mouse per lane). The 8‑hr time point was before ovulation; 15 hr was after ovulation, 48 hr was GD2; and 96 hr was GD4. *p < 0.05 compared with control.

Three of the most highly up-regulated genes on GD2 were the homeobox transcription factors *Pitx1*, *Nkx3-1* [NK-3 transcription factor, locus 1 (Drosophila)], and *Six1*. These genes all have critical roles in embryonic development but are not reported to be expressed in FRT tissues. We hypothesized that misexpression of these early development transcription factors contributed to abnormal oviduct morphogenesis in Gen-treated mice. To test this idea and to further verify the microarray data, we performed real-time RT-PCR on independently collected biological replicates. Oviducts were collected on PND5, on PND22 (prepubertal) from superovulated adult mice before and after ovulation, and on GD2 and GD4. *Pitx1* and *Nkx3-1* were highly up-regulated in Gen-treated mice compared with controls at all time points (Figure 3A,B). Pitx1 protein was not detected in control oviducts on PND5 (Figure 3C,E). However, Gen treatment induced Pitx1 expression in nuclei of sporadic oviduct epithelial cells in all areas of the PND5 oviduct (Figure 3D,F). As on PND5, Pitx1 was not observed in adult control oviducts (Figure 3G,I), but it was abundantly expressed in focal areas throughout the oviduct epithelium of adult Gen-treated mice (Figure 3H,J). Like *Pitx1*, *Six1* mRNA was highly up-regulated at all time points, and Six1 protein was significantly increased in the oviducts of Gen-treated mice compared with controls (Figure 3K,L).

**Figure 4:**
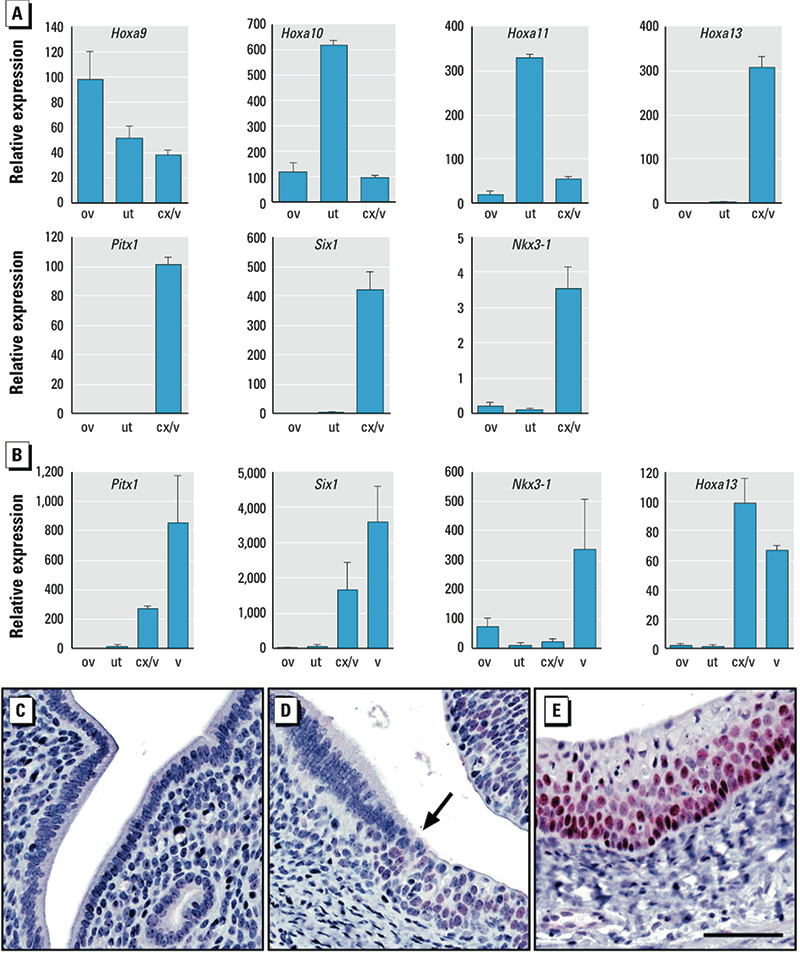
Expression of homeobox transcription factors in FRT tissues of control mice. (*A* and *B*) Relative expression (mean ± SE) of genes in tissues from PND1 (*A*) and adult (*B*) mice. (*C*–*E*) Pitx1 immunohistochemistry in adult uterus (*C*), cervix (*D*), and vagina (*E*). Bar = 50 μm. The arrow in *D* indicates squamocolumnar junction. Abbreviations: cx, cervix; ov, oviduct; ut, uterus; v, vagina.

*Broadly posteriorized oviduct gene expression after neonatal Gen treatment.* Because oviduct Hoxa gene expression was posteriorized in Gen-treated mice, we tested the idea that aberrant expression of *Pitx1*, *Six1*, and *Nkx3-1* was also related to FRT posteriorization. To do this, we first needed to determine the timing of Hoxa gene pattern establishment in controls. Hoxa gene patterning in PND1 controls resembled that in adult controls; that is, the highest levels of *Hoxa9* were in the oviduct, *Hoxa10* and *Hoxa11* in the uterus, and *Hoxa13* in the cervix/vagina (Figure 4A). These findings, together with alterations in the oviduct Hoxa pattern in Gen-treated mice on PND5 (Figure 2), indicate that Gen exposure disrupted maintenance of previously established Hoxa gene patterning.

To determine if genes highly up-regulated in the PND5 oviduct by Gen treatment are normally expressed in the posterior FRT, we compared gene expression levels in the oviduct, uterus, cervix, and vagina of control mice. *Pitx1*, *Six1*, and *Nkx3-1*, like *Hoxa13*, were expressed exclusively in the cervix/vagina and not in the uterus or oviduct on PND1 (Figure 4A) and PND5 (data not shown). This expression pattern was still observed in control adults, when *Pitx1*, *Six1*, *Nkx3-1*, and *Hoxa13* were highly expressed in the vagina and cervix but had minimal expression in the uterus or oviduct (Figure 4B). There was no Pitx1 protein in the adult uterus (Figure 4C). However, beginning in the cervix at the squamocolumnar junction, Pitx1 was detected in the nuclei of squamous epithelial cells (Figure 4D), and the staining intensity increased in vaginal epithelium (Figure 4E). Expression of the secreted signaling factors *Wnt4*, *Wnt5a*, *Wnt7a*, and *Fstl1* was similar in all regions of the FRT on PND1 and PND5 and in adults (data not shown). In contrast, several hedgehog pathway genes and *Foxa2* were more highly expressed on PND1 in the cervix/vagina compared with both the oviduct (*Ihh*, 11.0-fold; *Dhh*, 5.8-fold; *Hhip*, 42.1-fold; and *Foxa2*, 6.1-fold increased) and uterus (*Ihh*, 9.4-fold; *Dhh*, 6.2-fold; *Hhip*, 63.1-fold; and *Foxa2*, 651.7-fold increased), suggesting that these genes are also normally restricted to the posterior FRT. Together, these data demonstrate that neonatal Gen treatment blocked the normal process of transcriptional silencing of many posterior developmental genes in the anterior FRT.

**Figure 5:**
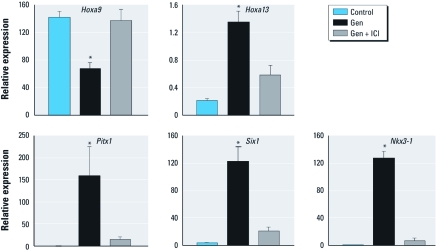
Inhibition of posteriorized gene expression (mean ± SE) by ER antagonist in the oviduct on PND5. *p < 0.05 compared with control or Gen + ICI.

To determine if the effects of neonatal Gen treatment on the oviduct are mediated by its estrogenic actions (Jefferson et al. 2002), we examined whether the estrogen receptor (ER) antagonist ICI (Wakeling and Bowler 1992) blocked these effects. Neonatal mice treated with both Gen and ICI did not have altered expression of *Hoxa9* and *Hoxa13*, nor did they have altered expression of the three most highly up-regulated homeobox transcription factors (*Pitx1*, *Six1*, and *Nkx3-1*) observed in mice treated with Gen alone (Figure 5). These findings indicate that molecular posteriorization of the oviduct in response to Gen treatment is mediated by its estrogenic action.

## Discussion

The results presented here demonstrate that a brief neonatal exposure to the phytoestrogen Gen causes substantial permanent changes in oviduct morphology and gene expression in mice. The altered expression of Hoxa and Wnt genes in the oviduct combined with aberrant expression of homeobox transcription factors that are normally expressed only in the posterior FRT likely account for abnormal oviduct differentiation and subsequent abnormal tissue architecture and function later in life.

Cellular differentiation of the FRT occurs during postnatal development in rodents, ruminants, nonhuman primates, and humans (Gray et al. 2001). Based on the presence of ERα in the FRT epithelium at birth, it is not surprising that an estrogenic compound administered during this period disrupts FRT morphogenesis and cellular differentiation. Indeed, numerous reports have described the effects of the potent synthetic estrogen DES on both prenatal and postnatal FRT development. Most of these studies have focused on the uterus; however, a few reports described the effects of DES on oviduct histopathology (Newbold et al. 1985). Furthermore, the doses of DES used had much higher estrogenic activity than that of estrogenic chemicals typically encountered in daily life. The neonatal Gen dosing strategy used in the present study results in a serum Gen concentration similar to that in human infants fed soy-based infant formula (Jefferson and Williams 2010). This model is also likely relevant to human exposure to low levels of other estrogenic environmental chemicals.

Prenatal DES treatment alters the expression pattern of Wnt genes in the FRT on GD19 (Suzuki et al. 2007). Similarly, in the present study, neonatal Gen treatment resulted in increased *Wnt5a* expression and significantly reduced *Wnt7a* expression in the oviduct on PND5, indicating that both prenatal and neonatal exposure to estrogenic compounds disrupts Wnt gene expression in the FRT. Stratified luminal epithelium is observed in the uterus of *Wnt7a*-null mice (Miller and Sassoon 1998), and in the present neonatal Gen model we found *Wnt7a* repression and a marked up-regulation in the oviduct of genes that are normally observed in stratified cervical/vaginal squamous epithelium. These findings support the idea that *Wnt7a* is required for maintenance of the columnar epithelium in the oviduct and uterus and that Gen-induced *Wnt7a* repression in the neonatal FRT may promote oviduct posteriorization. However, if reduction in Wnt7a were sufficient to promote a cervical/vaginal phenotype, we would expect that in control mice, *Wnt7a* expression would be lower in these tissues than in the uterus and oviduct. Instead, in controls during the neonatal period, *Wnt7a* is highly expressed in the cervix and vagina and is not differentially expressed compared with the uterus and oviduct. For this reason, the oviduct posteriorization in Gen-treated mice cannot be attributed to reduction in Wnt7a alone.

Hoxa genes direct AP patterning of the FRT and maintain a distinct spatial distribution in adults. From GD16.5 through GD19, *Hoxa9* is similarly expressed throughout the FRT, but *Hoxa10*, *Hoxa11*, and *Hoxa13* are differentially expressed along the AP axis, with *Hoxa10* and *Hoxa11* highest in the uterus and *Hoxa13* highest in the cervix and vagina (Ma et al. 1998; Suzuki et al. 2007). On the day of birth (PND1), we found that *Hoxa10*, *Hoxa11*, and *Hoxa13* had an expression pattern similar to that on E19, whereas *Hoxa9* was more highly expressed in the oviduct compared with the uterus and cervix/vagina. This Hoxa gene expression pattern is permanently maintained from the day of birth (PND1) forward because FRT tissues from PND14 and adult mice have a similar expression pattern (Taylor et al. 1997). Hoxa9 is not essential for FRT development because *Hoxa9*-null mice have normal fertility (Fromental-Ramain et al. 1996). In contrast, *Hoxa10*- and *Hoxa11*-null mice have abnormal FRT development and function (Benson et al. 1996; Gendron et al. 1997). *Hoxa13*-null mice have agenesis of the caudal portion of the Müllerian duct, and *Hoxd13* (homeobox D13)-null mice that also lack one copy of *Hoxa13* exhibit severe malformations of the vagina and external genitalia, suggesting critical roles for these Hox gene paralogs in posterior FRT development (Fromental-Ramain et al. 1996; Warot et al. 1997).

Neonatal Gen exposure alters Hoxa gene expression in the oviduct, with *Hoxa9*, *Hoxa10*, and *Hoxa11* expression significantly reduced compared with controls and *Hoxa13* increased. Similarly, *Hoxa10* and *Hoxa11* are reduced in the uterus after neonatal DES exposure (Block et al. 2000; Ma et al. 1998; Suzuki et al. 2007). Because the adult pattern of Hoxa gene expression is normally present at birth, these data suggest that Hoxa gene patterning is not irreversible once established but retains some plasticity in the neonatal period. This finding is consistent with the observation that in *Wnt7a*-null mice, *Hoxa10* and *Hoxa11* expression in the uterus is normal at birth but then gradually decreases (Miller and Sassoon 1998), suggesting that Wnt7a is required for maintenance of FRT Hoxa gene patterning.

*Hoxa13* up-regulation may contribute to neonatal Gen-induced posteriorization of the oviduct by inducing expression of several genes normally restricted to the cervix and vagina. This finding is in agreement with a mouse knock-in study in which replacement of the Hoxa11 homeodomain with the Hoxa13 homeodomain (A11^13hd/13hd^) resulted in posteriorization of the uterus to resemble a cervix/vagina–like structure (Zhao and Potter 2001). More than 30 genes normally expressed in the cervix and not in the uterus were expressed in the uterus of the A11^13hd/13hd^ mutants. One of these genes was the transcription factor *Six1*, suggesting that *Six1* is normally regulated by Hoxa13 via a mechanism involving the homeodomain. In the present study, *Six1* was highly up-regulated in the adult oviduct after neonatal Gen treatment, and we demonstrated that its up-regulation begins during the time of Gen treatment and remains stable through adulthood. This expression pattern correlates with the up-regulation of *Hoxa13* in the oviduct, suggesting that Hoxa13 expression in the anterior FRT is a proximal factor in altering cellular differentiation and causing phenotypic and molecular posteriorization of the oviduct.

One explanation for the altered expression of Hoxa genes in the oviduct is that Gen-induced activation of ER-mediated transcription disrupts global regulation of Hoxa genes in the FRT. Indeed, *Hoxa10* and *Hoxa11* are regulated by estrogen in the adult FRT (Taylor et al. 1998). Regulation of Hox gene expression during mammalian development is complex and involves regulatory RNAs and proteins whose activities orchestrate the establishment and subsequent maintenance of the AP Hox expression patterns (for review, see Chopra 2010). Because Hoxa patterning in the FRT is already established at birth, alterations in initiation factors cannot explain Gen-induced disruption. Instead, alterations in maintenance factors, such as polycomb and trithorax group proteins, that preserve the initial Hox gene expression pattern and help cells “remember” which regulatory domains to keep silent and which ones to keep active, are more likely candidates.

Although Hox genes may function upstream of *Pitx1*, *Six1*, and *Nkx3-1* after Gen treatment, the converse may also be true. These three genes have critical roles in directing embryonic development of tissues outside the FRT. Six1 regulates development of numerous tissues, including the eye, skeletal muscle, and posterior limbs (Christensen et al. 2008; Laclef et al. 2003). Nkx3-1 is important for prostate development and marks a stem cell population that functions during prostate regeneration (Sciavolino et al. 1997; Wang et al. 2009). Nkx3-1 was previously reported to be absent in the FRT, but tissues from the cervix or vagina were not examined (Sciavolino et al. 1997). Pitx1 is involved in development of anterior branchial arch structures and the hindlimb (Lamonerie et al. 1996; Lanctot et al. 1999; Szeto et al. 1999). Pitx1 acts upstream of Hox genes during hindlimb specification because misexpression of Pitx1 in the chick wing bud leads to increased expression of *Hoxc10* and *Hoxc11* and results in transformation into a more leglike structure (Logan and Tabin 1999). The permanent up-regulation of *Pitx1*, *Six1*, and *Nkx3-1* in the oviduct of Gen-treated mice, combined with the importance of these genes in development of many embryonic tissues, suggests that one or all of these homeobox transcription factors may contribute to oviduct posteriorization after Gen treatment. Because Six1 causes initiation and metastasis of several types of human cancer (Christensen et al. 2008), its permanent up-regulation could contribute to the observed increase in FRT cancers in neonatal Gen-treated mice (Newbold et al. 2001).

Infants fed soy-based formulas have high circulating levels of Gen (1.5–13.2 μM) (Cao et al. 2009). The subcutaneous injection model of Gen exposure used in the present study produces serum circulating levels similar to those of human infants (*C*_max_ = 6.8 μM in female pups). Because the primary exposure of humans is oral exposure to the glycosylated form, genistin, we previously compared a dose response of oral exposure to genistin with that of subcutaneous exposure to Gen in neonatal mice (Jefferson et al. 2009a). Oral exposure to genistin in neonatal mice on PND1–PND5 produces similar circulating levels of Gen as a subcutaneous injection (~ 80%) and similar effects on the developing reproductive tract, including multioocyte follicles in the ovary, abnormal estrous cycles, and reduced fertility. All of these Gen effects are correlated with the circulating level of Gen and the estrogenic activity (uterine wet weight increase) during the time of treatment and are not dependent on the route of administration or form of Gen given. Therefore, the subcutaneous model used in the present study is a valid model for human infant exposures to Gen.

## Conclusions

The findings reported here raise the possibility that exposure to low levels of estrogenic environmental chemicals or phytoestrogens during sensitive developmental windows can alter developmental programming of FRT tissues. These changes could contribute to improper function of the FRT later in life that manifests as an infertility phenotype. Should this phenomenon occur in humans, it would provide the mechanistic basis for some cases of unexplained infertility, which is the sole infertility diagnosis in a significant percentage of couples who undergo assisted reproduction treatment by *in vitro* fertilization. In addition, the permanent up-regulation of developmental transcription factors in the FRT could have long-term effects on its structure and function, including influencing the development of benign or malignant pathologies.

## Supplemental Material

(120 KB) PDF
