# Supplementary material for: Permanent Oviduct Posteriorization after Neonatal Exposure to the Phytoestrogen Genistein
Source: Environ Health Perspect. 2011 Aug 2;119(11):1575–82. doi: 10.1289/ehp.1104018 (PMC3226509; doi:10.1289/ehp.1104018)
Supplement: (120 KB) PDF [file ehp.1104018.s001.pdf]

Supplemental Material

Permanent Oviduct Posteriorization Following Neonatal Exposure to the Phytoestrogen Genistein

Wendy N. Jefferson[1], Elizabeth Padilla-Banks[1], Jazma Y. Phelps[1],

Kevin E. Gerrish[2] and Carmen J. Williams[1,*]

Table of Contents

Supplemental Material, Table 1 p.2

Supplemental Material, Table 2 p.3

Supplemental Material, Figure 1 p.4

Supplemental Material, Table 1. Microarray Validation

Gene	Gene Name	Array Fold Change (Gen/Control)	PCR Fold Change (Gen/Control)
Pitx1	Paired-like homeodomain transcription factor 1	99.30	>100*
Nkx3.1	NK-3 transcription factor, locus 1 (Drosophila)	5.90	10.10
Slc47a1	Solute carrier family 47, member 1	4.09	5.90
Igj	Immunoglobulin joining chain	3.80	4.10
Prlr	Prolactin receptor	2.90	3.50
Slpi	Secretory leukocyte peptidase inhibitor	2.10	2.60
Pgr	Progesterone receptor	-1.60	-1.50
Ihh	Indian hedgehog	-2.10	-2.00
Wnt7a	Wingless-related MMTV integration site 7A	-3.30	-3.00
Myh7	Myosin, heavy polypeptide 7, cardiac muscle, beta	-5.50	-5.30
Svs7	Seminal vesicle secretory protein 7	-8.50	-16.70
Ano2	Anoctamin 2	-18.40	-50.00

*Ct value >40 in all control samples; exact PCR fold change could not be calculated.

Supplemental Material, Table 2. Selected Ingenuity Biological Function Categories*

General Function	Category	# genes
Metabolism	Lipid Metabolism	29
	Carbohydrate Metabolism	39
	Amino Acid Metabolism	27
	Drug Metabolism	25
Hematological System	Hematological System Development and Function	74
	Hematopoiesis	25
	Morphology of Red Blood Cells	4
Immune Response	Immune Cell Trafficking	45
	Activation of B Lymphocytes	6
	Antigen Presentation	11
	Cell-mediated Immune Response	7
	Homing of Lymphatic System Cells	9
	Cytotoxicity of Leukocytes	11
Cell Proliferation	Cellular Growth and Proliferation	202
	Cell Morphology	130
	Cell Cycle	33
	Cellular Compromise	40
	Cell Death	215
Development	Endocrine System Development and Function	17
	Reproductive System Development and Function	22
	Renal and Urological System Development and Function	10
	Skeletal and Muscular System Development and Function	17
	Cardiovascular System Development and Function	61
	Connective Tissue Development and Function	16
	Lymphoid Tissue Structure and Development	21
	Embryonic Development	41

*Selected from biological function categories with all p-values < 0.025

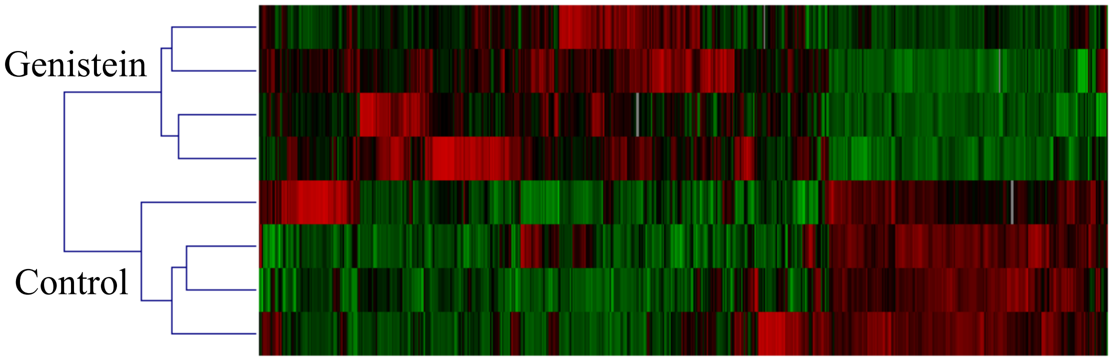

Supplemental Material, Figure 1. Hierarchical cluster analysis of differentially expressed oviduct genes in control and Gen-treated mice. Four biological replicates included per group. Red, upregulated genes; green, downregulated genes.
